# Vancomycin-induced nephrotoxicity in non-intensive care unit pediatric patients

**DOI:** 10.1038/s41598-021-00214-9

**Published:** 2021-10-19

**Authors:** Shinhyeung Kwak, Jeong Yeon Kim, Heeyeon Cho

**Affiliations:** Department of Pediatrics, Samsung Medical Center, Sungkyunkwan University School of Medicine, 81 Irwon-ro, Gangnam-gu, Seoul, 06351 South Korea

**Keywords:** Kidney diseases, Risk factors, Antimicrobials

## Abstract

Previous data suggested several risk factors for vancomycin-induced nephrotoxicity (VIN), including higher daily dose, long-term use, underlying renal disease, intensive care unit (ICU) admission, and concomitant use of nephrotoxic medications. We conducted this study to investigate the prevalence and risk factors of VIN and to estimate the cut-off serum trough level for predicting acute kidney injury (AKI) in non-ICU pediatric patients. This was a retrospective, observational, single-center study at Samsung Medical Center tertiary hospital, located in Seoul, South Korea. We reviewed the medical records of non-ICU pediatric patients, under 19 years of age with no evidence of previous renal insufficiency, who received vancomycin for more than 48 h between January 2009 and December 2018. The clinical characteristics were compared between patients with AKI and those without to identify the risk factors associated with VIN, and the cut-off value of serum trough level to predict the occurrence of VIN was calculated by the Youden’s index. Among 476 cases, 22 patients (4.62%) developed AKI. The Youden’s index indicated that a maximum serum trough level of vancomycin above 24.35 μg/mL predicted VIN. In multivariate analysis, longer hospital stay, concomitant use of piperacillin-tazobactam and serum trough level of vancomycin above 24.35 μg/mL were associated independently with VIN. Our findings suggest that concomitant use of nephrotoxic medication and higher serum trough level of vancomycin might be associated with the risk of VIN. This study suggests that measuring serum trough level of vancomycin can help clinicians prevent VIN in pediatric patients.

## Introduction

Vancomycin, a glycopeptide antibiotic, has been the first-line therapy for methicillin-resistant *Staphylococcus aureus* (MRSA) since it was discovered in 1952, and it is widely used to treat Gram-positive bacterial infections in patients who are allergic to penicillin^1^. However, vancomycin-induced nephrotoxicity (VIN) has been reported in pediatric and adult patients and presents a challenge for the broad use of vancomycin. Studies have suggested that vancomycin could induce nephrotoxicity by free radical production and subsequent oxidative stress and mitochondrial damage, but the exact mechanism has not been determined^2,3^. In pediatric patients, the incidence of VIN has not been well established, and the prevalence was reported to range from 2.4 to 27.2% depending on the study population^4–6^. It is presumed that the prevalence of VIN could differ depending on whether the study population is comprised of intensive care unit (ICU) patients, but there is little data on this specific issue. Previous data suggested several risk factors of VIN, including higher daily dose, prolonged duration of treatment, underlying renal disease, ICU admission, and concomitant nephrotoxic medications^5–10^. However, these predisposing factors differed depending on study population, and no study has analyzed the variables for non-ICU pediatric patients^5^.

Correlation between serum level of vancomycin and occurrence of VIN remains unclear. Many studies have not identified a significant relationship between serum trough level of vancomycin and development of nephrotoxicity. Several studies have reported that serum trough level of vancomycin ≥ 15 μg/mL is a risk factor for VIN in children as well as in adults^8,11^, but most of those studies included ICU patents, who generally are severely ill, compared with non-ICU patients and might have included additional risk factors for VIN. The 2011 Infectious Diseases Society of America guidelines for vancomycin use in pediatrics stated that a vancomycin trough level of 15–20 μg/mL could be targeted for patients with severe MRSA infections, such as osteomyelitis, infective endocarditis, meningitis, and bacteremia^1^. This recommendation is extrapolated from adult studies but lacks evidence in pediatric patients. There are limited data in pediatric patients to suggest that monitoring the serum trough level of vancomycin improves outcomes; additionally, it is not known whether the current recommendation for trough level of vancomycin is safe to prevent VIN and if the target is effective for treating patients with severe infection. The purpose of this study was to investigate the prevalence of VIN and associated risk factors and to analyze cut-off value of serum trough level of vancomycin to predict the occurrence of VIN in non-ICU pediatric patients.

## Methods

### Study population

This single-center, retrospective, observational study was conducted at Samsung Medical Center (Seoul, South Korea) from January 1, 2009, to December 31, 2018, by electronic medical record review. Pediatric patients (age of 3 months–19 years) that were admitted in the general ward and received vancomycin for more than 48 h were included. We excluded patients that had a baseline estimated glomerular filtration rate (eGFR) < 60 mL/min/1.73m^2^, underlying kidney disease, a history of renal replacement therapy at the time of admission, or incomplete data for serum creatinine or body measurements.

All data were obtained from electronic medical records and analyzed in accordance with the ethical principles for medical research involving human subjects established in the Declaration of Helsinki of 1975, and revised in 2000. The informed consent was waived by the Institutional Review Board (IRB) of Samsung Medical Center which approved this study (IRB number 2020-12-017). The following clinical data were collected; age, sex, height, weight, serum blood urea nitrogen (BUN) and creatinine levels, length of hospital stay in days, duration of vancomycin therapy, vancomycin trough level, underlying conditions, inotropic or vasopressor medication use during vancomycin therapy to assess the clinical severity, concomitant nephrotoxic medications including loop diuretics, piperacillin/tazobactam (PIP/TAZ), angiotensin converting enzyme inhibitors (ACEi), and angiotensin II receptor blockers (ARB) which are prescribed at least once during vancomycin therapy.

### Definitions

Baseline serum creatinine was defined as the value measured at the time before vancomycin therapy. The eGFR was calculated using the original Schwartz formula^12–14^. Acute kidney injury (AKI) immediately after or during the use of vancomycin was considered VIN. Kidney injury was classified based on the Pediatric Risk, Injury, Failure, Loss, End Stage Renal Disease (pRIFLE) criteria and AKI was defined as eGFR decreased over 50%, which can be presented as Injury or Failure in pRIFLE criteria^15^. The serum trough level of vancomycin initially was measured within 24 h after administration of vancomycin. The highest trough level measured during vancomycin therapy was collected and defined as the maximum vancomycin trough level.

### Monitoring vancomycin serum trough level

Our center guidelines require the serum trough level of vancomycin measurement to be captured just before the 5th dose administration or 24 h after the first administration of vancomycin. The clinical pharmacokinetic service (Abbottbase® Pharmacokinetic System, version 1.10) of the Center is used by physicians to discuss and adjust the dosage of vancomycin with clinical pharmacists based on patient-specific pharmacokinetic factors. Serum trough level of vancomycin is measured every 24 h.

### Statistical analysis

Statistical analysis was performed using SPSS version 27.0 software, and a *p* value < 0.05 was considered statistically significant. For bivariate analyses, the categorical variables were compared using χ2 or the Fisher’s exact test, and continuous variables were assessed using Student’s t-test and the nonparametric Wilcoxon rank-sum test, when appropriate. Multivariable logistic regression was applied to analyze the risk factors for VIN. Variables with a p-value less than 0.1 in univariate logistic regression and variables thought to be clinically meaningful were considered, and a final model was constructed through variable selection using the stepwise selection method. The cut-off value of maximum trough level of vancomycin to predict VIN occurrence was calculated by the Youden’s index.

### Ethical approval

The Institutional Review Board (IRB) of Samsung Medical Center approved this study (IRB number 2020-12-017).

## Results

### Demographic and clinical data and AKI prevalence

A total of 476 vancomycin-treated non-ICU pediatric cases was included in the analysis. There was a higher prevalence of male patients in this study (male-to-female ratio: 1.47), and the mean age at the start of vancomycin administration was 8.3 years. The mean baseline eGFR was 190.45 mL/min/1.73m^2^. The mean maximum trough level of vancomycin was 18.79 μg/mL. In 129 cases, concomitant nephrotoxic medications were used during vancomycin therapy. The most common underlying disease was hemato-oncologic disease (61.6%), and 14.9% patients had no underlying disease.

A total of 22 patients (4.6%) developed AKI during vancomycin treatment; 20 cases of Injury (4.2%), and 2 cases of Failure (0.4%). The prevalence of AKI according to the patient age was follows; < 1 year 10.5%, 1–2 year 4.2%, 2–3 year 7.7%, 3–4 year 3.5%, 4–5 year 2.8%, 6–7 year 13.0%, 9–10 year 13.3%, 11–12 year 4.2%, 13–14 year 5.3%, 14–15 year 13.0%, 18–19 year 3.7% and no AKI in 5–6, 7–9, 10–11, 12–13 and 15–18 year. Most of the VIN cases recovered to normal renal function, although one progressed to chronic kidney disease (CKD) stage 2. Patient demographics are summarized in Table 1.

**Table 1:** Patient demographic and clinical data.

Characteristics	Value
Total non-ICU, > 48 h. vancomycin, n	476
Male: Female (ratio)	283:193 (1.47)
Vancomycin start age, year, mean ± SD	8.29 ± 5.90
Baseline eGFR, mL/min/1.73m^2^, mean ± SD	190.45 ± 77.61
Length of hospital stay, day, mean ± SD	26.11 ± 34.32
Vancomycin therapy duration, day, mean ± SD	9.96 ± 8.72
Use of inotropic or Vasopressor medication, n (%)	13 (2.73)
**Concomitant medication, n (%)**	
Furosemide, n (%)	105 (22.06)
Piperacillin/tazobactam, n (%)	25 (5.25)
ACEi or ARB, n (%)	14 (2.94)
**Combined usage, n (%)**	
Double, n (%)	13 (2.73)
Triple, n (%)	1 (0.21)
**Underlying disease, n (%)**	
Previously healthy	71 (14.92)
Hemato-oncologic disease	293 (61.55)
Neurologic disease	31 (6.51)
Gastro-intestinal disease	24 (5.04)
Syndromic disease	19 (3.99)
Cardio-vascular disease	12 (2.52)
Immune deficiency	11 (2.31)
Endocrine disease	6 (1.26)
Allergic disease	4 (0.84)
Pulmonary disease	2 (0.42)
Autoimmune disease	2 (0.42)
Musculoskeletal disease	1 (0.21)

*ICU* intensive care unit, *SD* standard deviation, *eGFR* estimated glomerular filtration rate, *ACEi* angiotensin converting enzyme inhibitors, *ARB* angiotensin II receptor blocker.

### VIN group comparisons

The results for the VIN group and group without VIN are shown in Table 2. The following variables showed significant association with the occurrence of VIN: longer duration of vancomycin therapy (13.7 days vs. 9.8 days, *p* = 0.018), greater number of days of hospitalization (45.4 days vs. 25.2 days, *p* = 0.0001), higher maximum vancomycin trough level (23.5 μg/mL vs. 18.6 μg/mL, *p* = 0.013), and concomitant use of nephrotoxic medications (furosemide; 40.9% vs. 21.2%, *p* = 0.0367, PIP/TAZ; 22.7% vs. 4.4%*, p* = 0.0038). There was no concomitant use of inotropic or vasopressor medication during vancomycin therapy in the VIN group.

**Table 2:** Clinical characteristics of patients with and without VIN.

Variable	AKI (*n* = 22)	Non-AKI (*n* = 454)	*p*-value
Male: Female (ratio)	14:8 (1.75)	269:185 (1.45)	0.6824
Age at vancomycin treatment (years)^a^	6.67 ± 5.71	8.37 ± 5.91	0.143
Length of hospital stay (days)^a^	45.36 ± 42.46	25.18 ± 33.65	0.0001
Baseline eGFR (mL/min/1.73m^2^)^a^	282.62 ± 127.66	185.99 ± 71.61	< 0.0001
Duration of vancomycin therapy (days)^a^	13.73 ± 9.70	9.78 ± 8.64	0.018
Duration from initial vancomycin administration to initial trough level (hours)^a^	27.16 ± 13.56	30.5 ± 22.31	0.4297
Duration from initial vancomycin administration to maximum trough level (hours)^a^	178.79 ± 154.02	125.01 ± 117.18	0.1122
Initial trough level of vancomycin (μg/mL)^a^	15.10 ± 12.84	10.94 ± 9.85	0.0687
Maximum trough level of vancomycin (μg/mL)^a^	23.46 ± 11.30	18.56 ± 12.28	0.013
Use of inotropic or Vasopressor medication, n (%)	0	13 (2.86)	
**Concomitant medication, n (%)**			
Furosemide, n (%)	9 (40.91)	96 (21.15)	0.0367
Piperacillin/tazobactam, n (%)	5 (22.73)	20 (4.41)	0.0038
ACEi or ARB, n (%)	1 (4.55)	13 (2.86)	0.4893
Combined usage, n (%)	1 (4.55)	13(2.86)	0.4893
Double, n (%)	1 (4.55)	12 (2.64)	
Triple, n (%)	0	1 (0.22)	
**Underlying disease, n (%)**			
Previously healthy	1 (4.55)	70 (15.42)	
Hemato-oncologic disease	11 (50)	282 (62.11)	
Neurologic disease	2 (9.09)	29 (6.39)	
Gastro-intestinal disease	2 (9.09)	22(4.85)	
Syndromic disease	2 (9.09)	17 (3.74)	
Cardio-vascular disease	1 (4.55)	11 (2.42)	
Immune deficiency	1 (4.55)	10(2.2)	
Endocrine disease	2 (9.09)	4 (0.88)	
Allergic disease	0	4 (0.88)	
Pulmonary disease	0	2 (0.44)	
Autoimmune disease	0	2 (0.44)	
Musculoskeletal disease	0	1(0.22)	

^a^Data are presented as the mean with standard deviation.

*VIN* vancomycin-induced nephrotoxicity, *AKI* acute kidney injury, *eGFR* estimated glomerular filtration rate, *ACEi* angiotensin converting enzyme inhibitors, *ARB* angiotensin II receptor blocker.

### Contributing factors for VIN

The number of days of hospitalization, duration of vancomycin therapy, concomitant use of furosemide or PIP/TAZ, and vancomycin trough level were associated independently with the occurrence of VIN (Table 3). Multivariate logistic regression analysis showed that longer hospital days (OR 1.008, *p* = 0.0434), a maximum vancomycin trough level over 24.35 μg/mL (OR 3.519, *p* = 0.006) and concomitant use of PIP/TAZ (OR = 4.407, *p* = 0.0128) increased the risk of VIN (Table 4).

**Table 3:** Univariate logistic regression analysis of independent risk factors for VIN.

Variable	OR	95% CI	*p* value
Hospital day (day)	1.009	1.002–1.017	0.0149
**Concomitant nephrotoxic agents**			
Furosemide	2.582	1.072–6.220	0.0345
Piperacillin/tazobactam	6.382	2.139–19.045	0.0009
Combined administration	1.615	0.202–12.937	0.6514
Vancomycin treatment duration (day)	1.034	1.000–1.069	0.0474
**Vancomycin trough concentration**			
Maximum trough level ≥ 24.35 μg/mL	3.83	1.611–9.105	0.0024
Initial trough level (μg/mL)	1.021	0.996–1.046	0.0984
Maximum trough value (μg/mL)	1.02	0.997–1.042	0.0873
Average trough value over 72 h (μg/mL)	1.053	0.997–1.113	0.0616

*VIN* vancomycin-induced nephrotoxicity, *OR* odds ratio, *CI* confidence interval, *BUN* blood urea nitrogen. Continuous variable odds ratio is odds per single unit indicated in parenthesis.

**Table 4:** Multivariate logistic regression analysis of risk factors for VIN.

Variable	OR	95% CI	*p* value
Hospital days (day)	1.008	1.000–1.017	0.0434
Concomitant Piperacillin/tazobactam	4.407	1.370–14.170	0.0128
Maximum vancomycin trough concentration ≥ 24.35 μg/mL	3.519	1.434–8.632	0.0060

*VIN* vancomycin-induced nephrotoxicity, *OR* odds ratio, *CI* confidence interval. Continuous variable odds ratio is odds per single unit indicated in parenthesis.

### Optimal vancomycin trough level for AKI prediction

**Figure 1:**
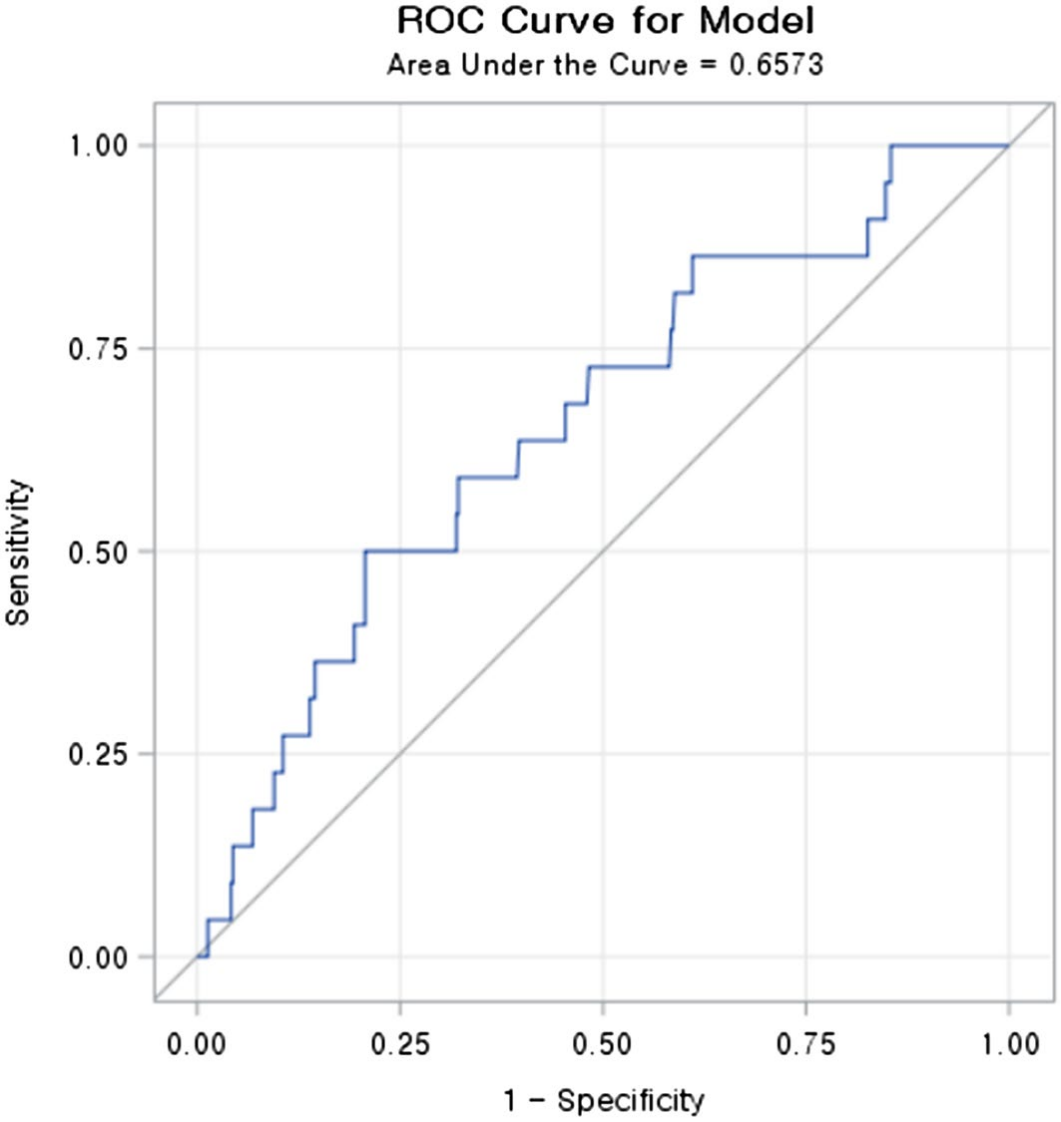
ROC curve to estimate the optimal cut-off value for maximum trough level of vancomycin (AUC = 0.6573).

To predict VIN in non-ICU pediatric patients, the optimal cut-off value for the maximum vancomycin trough level was estimated by Youden’s index. A maximum trough level of vancomycin above 24.35 μg/mL predicted VIN in non-ICU children (sensitivity 50%, specificity 79.3%, accuracy 77.9%). Receiver operating characteristic (ROC) curve analysis indicated that the area under the ROC curve (AUC) was 0.6573 (95% confidence interval 0.5394–0.7752, *p* = 0.0089) (Fig. 1).

## Discussion

This study evaluated the prevalence and associated risk factors of VIN in non-ICU pediatric patients. Previous studies have reported a wide range of VIN incidence, from 2.4 to 27.2%^4–6^, which is likely due to clinical differences in the study population, as well as heterogeneity in vancomycin dosing and the definition of VIN^5,7,16,17^. In this study, AKI occurred in 4.6% of 476 vancomycin-treated non-ICU pediatric patients, which was relatively low compared to previous reports. This difference is likely because our study was performed to assess non-ICU pediatric patients with relatively stable vital signs.

In this study, PIP/TAZ use independently increased the risk of VIN occurrence, which is consistent with previous reports^5,11,16,18–20^. A previous study indicated that children with renal insufficiency were more likely to receive furosemide compared with children with normal kidney function^11^. The risk of VIN in children that receive furosemide might be attributable to baseline renal insufficiency and to the additional nephrotoxicity caused by furosemide. For patients that received PIP/TAZ, it is unclear whether the risk of VIN is due to the severity of infection or synergistic drug toxicity. We also investigated whether the use of ACEi or ARB, and inotropic or vasopressor medications could influence the occurrence of VIN and determined that there was no association between the occurrence of VIN and the use of these medications in non-ICU pediatric patients.

Vancomycin trough level has been evaluated commonly as a potential predictor of nephrotoxicity in many previous studies. Several studies in adults have reported that the risk of AKI increases as the trough level of vancomycin increases^8,21^, but data in children are limited. While the guidelines recommend vancomycin trough level of 15–20 μg/mL to achieve more effective overall antibiotic exposure in severe infectious diseases, a few studies in pediatric patients have assessed the safety of this recommendation by comparing the incidence of nephrotoxicity of children with values above these ranges and those with values below 15 μg/mL^5,6,11^. The ideal therapeutic trough level for preventing nephrotoxic AKI has not been confirmed. In this study, the cut-off value of trough level to predict nephrotoxicity was calculated by Youden's J statistical analysis. When this estimated cut-off value was applied, both univariate and multivariate logistic regression analyses showed that vancomycin trough level ≥ 24.35 μg/mL is a risk factor for VIN. With the limitation of retrospective study design of this study, we do not know the causal relationship between the over 24.35 μg/mL vancomycin trough level and AKI. However, this finding suggests that regular monitoring of vancomycin trough level and effort to keep the level below 24.35 μg/mL could be helpful to prevent VIN in non-ICU pediatric patients. Despite the low value of AUC 0.6573, which indicates the risk of low accuracy, the negative predictive value was 97.04%, which suggested that the risk of AKI could be reduced under conditions where the serum trough level is below 24.35 μg/mL. Moreover, according to these results, targeting a vancomycin trough level of 15–20 μg/mL for severe infections, as indicated in the current guidelines, could be justified for non-ICU pediatric patients without underlying kidney disease. Furthermore, in the previous reported meta-analysis, the diagnostic accuracy analysis of AKI according to vancomycin trough value demonstrated AUC 0.681 and 0.637 in vancomycin trough level of 15 and 20 μg/mL, respectively^22^. These consistently low AUC values, including the result from this study, might indicate the presence of the unobserved variable, which need to be revealed in future research.

Previous studies have shown that the number of days in hospital and duration of vancomycin treatment are related to occurrence of AKI^5,7^. In our study, the total durations of hospitalization and vancomycin therapy were significantly longer in the group with nephrotoxicity than in the group without nephrotoxicity. However, in multivariant analysis, only duration of hospitalization was associated to VIN. It is possible that the longer hospitalization and prolonged vancomycin use could be related to disease severity and highly influenced by many other variables.

Even though most cases of AKI that are induced by vancomycin treatment recovered in our study, one patient progressed to CKD stage 2. The case with CKD was a 6-month-old boy that had underlying disease with biliary atresia and was treated with vancomycin for 10 days with concomitant use of furosemide.

With aforementioned limitation of retrospective study, we do not know the causal relationship between risk factors revealed in this study and VIN. However, for the non-ICU pediatric patients with the VIN risk contributing factors, clinicians could have chance to re consider alternative antibiotics for gram-positive bacterial infection such as teicoplanin which is known to be less nephrotoxic and similar effect compare to vancomycin^23^.

Our study had some limitations. First, more than half of the patients in this study had underlying disease with malignancy, and their renal function could have been impacted because of the previous exposure to nephrotoxic agents. Second, this study was a single center and retrospective study in which it was difficult to control the actual time of blood sample collection. Third, the low prevalence of VIN in this study might have impacted the power of statistical analysis. In order to minimize the bias (under- and over-estimation) of multiple logistic regression, variable selection was required to create a final model consisting of only important variables, and a variable selection method called stepwise selection method was used. For validation, a further multicenter large sample study could be helpful. Finally, several factors that could affect nephrotoxicity, such as intravenous contrast dye were not evaluated because of the limited available data.

## Conclusion

The prevalence of VIN in non-ICU pediatric patients was 4.6% in our study, and concomitant nephrotoxic agents such as PIP/TAZ and higher serum trough level of vancomycin were risk factors for VIN. Therefore, predicting the risk for AKI based on the highest trough level of vancomycin can help clinicians identify and prevent VIN in non-ICU pediatric patients.

## Data Availability

The datasets generated during and/or analysed during the current study are available from the corresponding author on reasonable request.

## Code availability

Statistical analysis was performed using SPSS version 27.0 software.
